# CD40/CD40L expression and its prognostic value in cervical cancer

**DOI:** 10.1590/1414-431X2023e13047

**Published:** 2023-11-13

**Authors:** G.A. Grazia, D.R. Bastos, L.L. Villa

**Affiliations:** 1 Departamento de Radiologia e Oncologia, Faculdade de Medicina, Universidade de São Paulo, São Paulo, SP, Brasil; 2 Centro de Investigação Translacional em Oncologia, Instituto do Câncer do Estado de São Paulo, São Paulo, SP, Brasil

**Keywords:** CD40, CD40L, Cervical cancer, SiHa, HeLa

## Abstract

CD40, a member of the tumor necrosis factor receptor (TNFR) family, is known to be involved in immune system regulation, acting as a costimulatory molecule, and in antitumor responses against cancer cells. It is a protein that is expressed in different types of cells, including immune cells and cancer cells (e.g., cervical cancer, breast cancer, melanoma). In this study, we investigated CD40/CD40L transcriptional and protein levels in cervical cancer cell lines and tumors. Higher CD40 expression was observed in cervical cancer cell lines derived from squamous cell carcinomas than from adenocarcinomas. Search of CD40/CD40L expression in cervical cancer tissues in public data sets revealed that about 83% of squamous cell carcinomas express CD40 compared to other cervical tumor subtypes. Moreover, expression of CD40 and CD40L in squamous cervical carcinomas is associated with better overall survival. Therefore, these proteins could be explored as prognostic markers in cervical cancers.

## Introduction

Cervical cancer is the fourth most common type of cancer in women worldwide, with about 604,000 new cases and 342,000 deaths in 2020, of which 90% are in developing countries. Moreover, this tumor is associated to persistent infection with high-risk human papillomavirus (HPV) (1).

Understanding cervical cancer development is central to diagnostic and treatment strategies. The CD40 pathway and its activation can exert different roles in cancer, where it can lead to apoptotic or pro-survival effects (2). The interaction between CD40 and its ligand CD40L can cause tumor growth inhibition due to immunologic mechanisms and apoptosis induction; alternatively, it may promote tumor growth through the action of cytokines and growth factors, such as interleukin 6 and vascular endothelial growth factor (3).

CD40 belongs to the tumor necrosis factor family and it is a type I transmembrane glycoprotein. It is expressed in different types of cells, including antigen-presenting cells, fibroblasts, epithelial cells, and tumors cells (4-6). The CD40 ligand, CD40L, a type II membrane protein, also a member of the tumor necrosis factor family, is expressed on monocytes, B and T cells, epithelial cells, fibroblasts, and others (7).

The interaction between CD40 and CD40L can activate dendritic cells, improving their antigen presentation capacity. Consequently, it may lead to upregulation of other costimulatory molecules (e.g., major histocompatibility complex II (MHC II) and CD80/CD86) and downregulation of immunosuppressive molecules. Moreover, a pro-survival signal and an increase of cytokines release culminate in higher cytotoxic response and prevention of immune tolerance induction (2).

CD40 expression can have different consequences depending on tumor type. In breast cancer cell lines, Tong et al. examined CD40 expression and the growth-inhibitory effects of CD40L (8). In the study conducted by Weiss et al. (9) CD40 expression indicated a favorable prognostic effect in renal cell carcinoma. On the other hand, in lung cancer, CD40 is correlated with metastasis and poor prognosis (10).

To better understand the relationship between CD40 and cervical cancer, we evaluated CD40 expression in cervical cancer cell lines at the RNA and protein levels. Furthermore, we analyzed CD40 and CD40L expression in cervical cancer tissues and their prognostic value based on public databases.

## Material and Methods

### Cell culture

Primary foreskin keratinocytes (PHK; Lonza, Switzerland) were grown in Keratinocyte Basal Medium (KGM-Gold^TM^; Lonza) supplemented with growth factors (Keratinocyte Growth Medium SingleQuots™; Lonza). The following cervical cancer-derived cell lines were employed: C33A (HPV-negative; ATCC^®^ CRM-HTB-31™), SiHa (HPV16; ATCC^®^ HTB-35™), SW756 (HPV18; ATCC^®^ CRL-10302^TM^), and HeLa (HPV18; ATCC^®^ CCL-2™); all cell lines were grown in MEM (Gibco^TM^, Invitrogen, USA) with 10% fetal bovine serum. The cell line BT-549 (ATCC^®^ HTB-122™), MDA-MB-453 (ATCC^®^ HTB-131™), MDA-MB-231 (ATCC^®^ HTB-26™), and SKBR3 (ATCC^®^ HTB-30™) were grown in RPMI-1640 culture medium (Gibco), supplemented with 10% fetal bovine serum (FBS) (Gibco). All cell lines used have been authenticated and tested for mycoplasma. They were maintained at 37°C and 5% CO_2_ (Thermo Electron Corporation, USA).

### RNA isolation and reverse transcription-polymerase chain reaction (RT-PCR)

RNAs derived from the cell cultures were extracted using TRIzol reagent (Invitrogen), following the manufacturer's instructions. RNA samples were treated with DNAase RQ1 (Promega Corp., USA). cDNA was synthesized using Go Script^TM^ Reverse Transcription System Kit (Promega Corp.). RT-PCR reactions were carried out using Go Taq^®^ qPCR Master Mix (Promega Corp.). Primers specific for CD40, CD40L, and for the housekeeping gene GAPDH were synthesized by Thermo Fisher Scientific (USA). Primer sequences (from 5′ to 3′) were: human CD40 forward: GGCAGGCACAAACAAGACTG, human CD40 reverse: GGCAAACAGGATCCCGAAGA, human CD40L forward: GCAAATACCCACAGTTCCGC; human CD40L reverse: GACAAACACCGAAGCACCTG and human GAPDH forward GACTGTGGTCATGAGTCCTCCC, GAPDH reverse: CAAGATCATCAGCAATGCCTCC. The RT-PCR reactions were processed in an ABI Prism 7500 equipment (Applied Biosystems, USA), and the delta-delta CT method was applied for quantification.

### Protein extraction and western blotting

Whole cell lysates were extracted using protein extraction buffer (150 mM NaCl, 50 mM Tris-HCl pH 7.5, 0.5% NP40, and 0.1 mM EDTA), supplemented with a cocktail of protease and phosphatase inhibitors (#11836153001, Complete Mini Protease Inhibitor, Roche Diagnostics, Switzerland). The extracts were quantified using the Bradford protein assay (#5000006, BioRad Protein Assay Dye, BioRad, USA) in a Benchmark Microplate Reader (iMark^TM^ Microplate Reader, BioRad). Then, 50 μg of protein was separated by SDS-PAGE and transferred (Bio-Rad Power PAC 300) to polyvinylidene difluoride (PVDF) membranes (Hybond-P, GE Healthcare, UK). Next, the membranes were blocked in 5% non-fat dry milk in PBS-T (137 mM NaCl, 2.7 mM KCl, 10 mM Na_2_HPO_4_, 2 mM KH_2_PO_4_ pH=7.4, and 0.1% Tween-20) for two hours and immunoblotted with specific antibodies (CD40 - MAB6322-SP - R&D, Biotechne, USA; CD40L - MA532619, Invitrogen, Thermo Fisher Scientific, USA; GAPDH - Ab181602, Abcam, UK; Tubulin - Ab52866, Abcam). The detection of the proteins was performed by chemiluminescent method, using ECL^TM^ Prime Western Blotting Detection Reagent kit (#RPN2232, GE Healthcare Life Sciences, UK), and the images were acquired on the ImageQuant LAS 4000 (GE Healthcare Life Sciences) and analyzed with ImageJ software (version v1.53t, 2022, NIH, USA).

### *In silico* analysis of CD40/CD40L gene expression

The cBioPortal online platform (https://cbioportal.org) contains gene expression databases from patients with different types of cancer (11,12). In the present study, we assessed the information of CD40 and CD40L from 310 patients with cervical cancer. Cases without information of gene expression were excluded. The clinical information was cross-referenced with quantitative and qualitative expression data for associations and correlation statistics. The CD40 and CD40L was expressed as log-transformed mRNA expression z-scores compared to the expression distribution of all samples (RNA Seq V2 RSEM). To analyze CD40 and CD40L prognostic associations, the KMPlotter platform (kmplot.com/analysis/) was used (13). We conducted tests to evaluate overall survival (OS) and relapse-free survival (RFS).

### Statistical analysis

All analyses were conducted with the Statistical Package for Social Sciences (SPSS Statistics 25.0; IBM, USA) or GraphPad 8.1.1 (GraphPad, USA). The Gaussian distribution analysis of the groups was tested by the Kolmogorov-Smirnov method. The chi-squared or Fisher's exact test was applied to compare categorical variables. The comparison between two groups was performed using the Student's *t-* or Mann-Whitney test, and for three or more groups we used ANOVA or the Kruskal-Wallis test. Correlations between CD40 and CD40L gene expression levels were evaluated by Pearson's correlation. Survival analysis was conducted using the KMPlotter platform, which uses the Kaplan-Meyer method with logrank test and Cox regression. Statistical significance was considered at a P-value <0.05.

## Results

### Different levels of CD40 were expressed by cervical cancer cell lines

**Figure 1:**
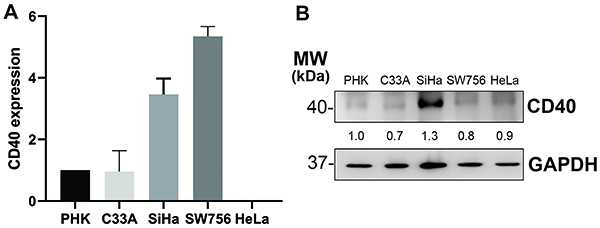
CD40 expression in cervical cancer cell lines. **A**, RT-PCR analysis of CD40 RNA in cervical cancer cell lines and (**B**) western blot analysis of CD40 protein levels in cervical cancer cells lines. PHK: primary human foreskin keratinocytes.

CD40 RNA levels were higher in SiHa and SW756 cell lines (Figure 1A). On the other hand, no CD40 RNA was detected in HeLa cells. Concerning protein levels, we observed that SiHa presented higher CD40 protein levels compared to the other cell lines (Figure 1B). Interestingly, SW756 did not show high levels of CD40 protein despite very high levels of CD40 RNA. We have also tested both RNA and protein levels of CD40L and found no expression in the selected cell lines (Supplementary Figure S1).

### High CD40 expression was associated with good prognosis in cervical cancer

cBioPortal contains different microarray and RNAseq databases, including the TCGA Firehose Legacy project. Samples without quantification of CD40 or CD40L were excluded from the analysis. The contingency table (Table 1) points to significant differences between CD40 and patient's age and cancer type and staging. None of the variables were associated with different expression levels of CD40L.

**Table 1:** Clinical-pathological characteristics from TCGA Firehose Legacy cervical cancer patients.

Parameters	CD40			CD40L		
	Lown (%)	Highn (%)	P	Lown (%)	Highn (%)	P
Age (years)						
≤50	105 (55.9)	83 (44.1)	0.011*	100 (53.8)	86 (46.2)	0.111
>50	47 (40.9)	68 (59.1)		50 (44.2)	63 (55.8)	
Cancer Type						
SCC	113 (44.8)	139 (55.2)	<0.0001*	124 (49.8)	125 (50.2)	0.818
Adeno	22 (84.6)	4 (15.4)		12 (48)	13 (52)	
Others	17 (68)	8 (32)		14 (56)	11 (44)	
Stage						
I/II	105 (46.1)	123 (53.9)	0.013*	111 (49.3)	114 (50.7)	0.757
III/IV	43 (63.2)	25 (36.8)		35 (51.5)	33 (48.5)	
NHG						
1	10 (52.6)	9 (47.4)	0.851	11 (57.9)	8 (42.1)	0.651
2	65 (48.9)	68 (51.1)		63 (48.1)	68 (51.9)	
3	62 (52.5)	56 (47.5)		61 (52.1)	56 (47.9)	
4	0 (0)	1 (100)		0 (0)	1 (100)	
Menopause status						
Pre	72 (57.1)	54 (42.9)	0.075	66 (53.2)	58 (46.8)	0.291
Peri	14 (56)	11 (44)		15 (60)	10 (40)	
Pos	33 (41.3)	47 (58.8)		35 (44.3)	44 (55.7)	

Data are reported as number and percent. SCC: cervical squamous carcinoma; Adeno: adenocarcinoma; Others: includes mucinous carcinoma, cervical endometrioid carcinoma; NHG: neoplastic histologic grade. Patients were stratified into the high or low category by the median CD40 and CD40L. *P<0.05, chi-squared test or Fisher's exact test.

**Figure 2:**
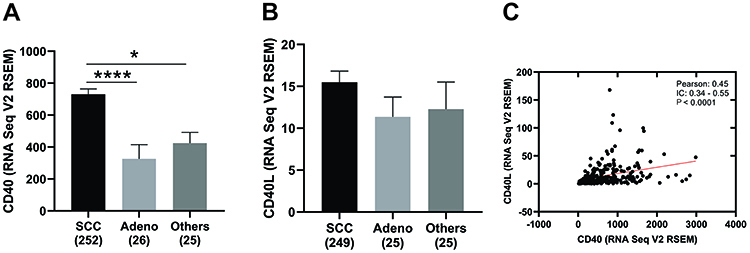
CD40 and CD40L RNA levels in cervical cancer by histological subtype. **A**, CD40 mRNA expression in squamous cervical carcinomas (SCC), adenocarcinomas (Adeno), and other subtypes. ****P<0.0001 and *P<0.05, Kruskal-Wallis test was applied with Dunn's multiple comparison test. **B**, CD40L mRNA expression in SCC, Adeno, and other subtypes. **C**, Correlation between CD40 and CD40L RNAseq transcript levels based on TCGA cervical cancer data set (P<0.0001, Spearman test). The analyses are based on TCGA Firehose Legacy cervical cancer patients. CD40L: CD40 ligand.

A significantly higher CD40 expression was observed in cervical cancers of the squamous cell subtype than adenocarcinomas or other histological tumor types (Figure 2A). On the other hand, no difference was observed related to CD40L in these subtypes (Figure 2B). It is noteworthy that a positive correlation was observed between CD40 and CD40L transcript levels as inferred from the RNAseq analysis of cervical squamous carcinomas (Figure 2C).

**Figure 3:**
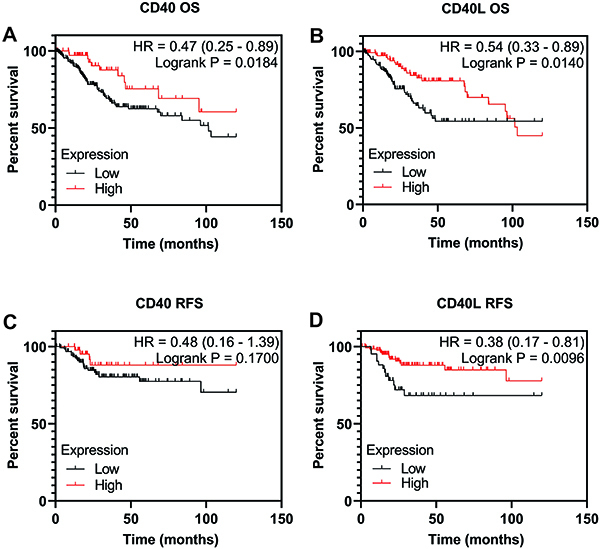
Survival analysis in cervical cancer patients by levels of CD40 and CD40L. Overall survival (OS) of (**A**) cervical squamous carcinoma patients according to CD40 expression and (**B**) cervical squamous carcinoma patients according to CD40L expression. Relapse-free survival (RFS) of (**C**) cervical squamous carcinoma patients according to CD40 expression and (**D**) cervical squamous carcinoma patients according to CD40L expression. The analyses are based on TCGA Firehose Legacy cervical cancer patients. The Kaplan Meier method with log-rank test was applied. CD40L: CD40 ligand.

We also verified if CD40 and CD40L had a prognostic role in cervical squamous carcinomas through a KMPlotter analysis (13) in the same population from the TCGA Firehose Legacy project, as depicted in Figure 3. Interestingly, patients with low expression of CD40 or CD40L presented worst OS in 120 months (Figure 3A and B). For RFS, only CD40L low expression was associated with patient's poorer prognosis (Figure 3D). We also stratified patients by tumor staging and by the median expression of CD40 and CD40L, as depicted in Supplementary Figure S3. A lower OS was found in the group with advanced stages and low CD40/CD40L (Supplementary Figure S3A and B). As for RFS, no significant differences were observed (Supplementary Figure S3C and D).

## Discussion

CD40 is known to be a costimulatory molecule, involved in humoral and cell-mediated immune responses (14,15). Moreover, the CD40 pathway is associated with several diseases, including cancer, where the interaction between CD40 and CD40L is pleiotropic and context-dependent (3,14,16,17).

In this study, we aimed to examine CD40 expression in different cervical cancer cell lines at both RNA and protein levels and analyzed CD40 expression and its prognostic role in cervical cancer patients. We observed that SiHa (HPV16) and SW756 (HPV18) cell lines presented higher transcriptional levels of CD40 expression (Figure 1A) than normal keratinocytes (PHK) and HPV-negative cervical cancer cell line, C33A. Our result regarding normal keratinocytes corroborated the results of Denfeld et al. (18), which showed that human keratinocytes present low CD40 expression. Only SiHa exhibited higher CD40 protein expression (Figure 1B), suggesting that a post-transcriptional regulation may be associated to SW756 cell line due to its high transcriptional level and low protein expression level. In addition, we observed that none of these cell lines derived from cervical cancer expressed CD40L (Supplementary Figure S1). It is important to mention that different HPV types, such as HPV16 and HPV18, can interfere in the expression of different genes. That is the case of *FANCI-2* gene, for example, which its lncRNA expression increases as the pre-neoplastic lesions progress to cervical cancer, especially in the presence of HPV16 (19). The TCGA database for cervical cancer population shows a variety of HPV types, HPV16 being the most prevalent, followed by HPV18 and HPV45, and less frequently, HPV 30, 31, 33, 35, 39, 45, 52, 56, 58, 59, 68, 69, 70, and 73 (20). This heterogeneity of distinct HPV types was analyzed regarding CD40 expression (Supplementary Figure S2), which indicated that different HPV types may regulate CD40 expression in different ways.

Over the years, CD40 expression was the subject of several investigations. CD40 is a receptor expressed in a wide variety of cells and tissues, and the expression varies between 35-100% in established cell lines and depends on tumor histopathological type. In biopsies of melanoma and other epithelial tumors, CD40 expression is found in over 60% of the samples (21). The CD40 expression and function on epithelial cells will determine their role as immune effectors depending on the respective microenvironments, since CD40 activates these epithelial cells and promotes the release of pro-inflammatory, anti-inflammatory, and pro-fibrotic mediators (22). Moreover, CD40 is also detected in different cancer cells, whereas its ligand, CD40L, is hardly found in normal or malignant epithelial cells. This occurs because CD40L is primarily detected in the cytoplasm of cancer cells and rarely in their membrane, indicating that membrane-bound CD40L expression may be uncommon or transient, escaping detection (8,23). Furthermore, CD40 can be upregulated by IFN-γ in normal keratinocytes and in HPV-transformed cell lines. The CD40 ligation have a direct influence in the susceptibility of cervical carcinoma cells to cytotoxic T lymphocyte (CTL)-mediated killing. When CD40 from keratinocytes interacts with CD40L from Th1 cells, it increases ICAM-1 expression and stimulates the expression of cytokines and chemokines, promoting the activation and additional recruitment of immune cells (24).

Based on the review conducted by Moerman-Herzog and Nakagawa (24), we gathered important information about CD40 ligation on normal keratinocytes or on cervical cancer cells, showing that this ligation amplifies inflammatory reactions due to chemokine secretion to recruit leukocytes. Interestingly, this chemokine secretion is depressed by HPV. Cervical carcinoma cells have been shown to present a lower CCL2 secretion in response to CD40 ligation compared to non-tumorigenic HPV-positive cells. However, the combination of IFN-γ and CD40 ligation promotes a synergetic increase of CCL2 secretion in both cell types and a more pronounced synergistic upregulation of CXCL10. Another important point is that CD40 ligation is capable of producing defined networks of gene expression, which are coordinated by high expression of TNF and IL-8. In the case of HPV-positive epithelial cells, CD40 ligation promoted a similar network of gene expression. Moreover, CD40 ligation was described to increase even more in HPV-positive cells compared to uninfected epithelial cells, showing that despite the deficits in gene expression, CD40 ligation is able to partially reverse this process. Finally, previous data obtained by ELISA tests showed that HPV-positive cells secreted lower levels of CXCL8, CXCL9, CXCL10, and RANTES compared to uninfected epithelial cells in response to IFN-γ stimulation and CD40 ligation. In addition, supernatants of uninfected epithelial cells had the ability to increase peripheral blood mononuclear cell migration due to IFN-γ stimulation and CD40 ligation. Altogether, these *in vivo* events in epithelial cells may have an impact in HPV persistence and disease progression.

Here, we found that CD40 RNA levels were higher in squamous cell carcinomas than in other histological subtypes. This is in agreement with the high levels of CD40 protein expression in cervical cancer tissues (25). In addition, tissue samples derived from HPV-positive lesions and pre-neoplastic and malignant samples also express higher levels of CD40 compared to normal cervical epithelia. Another study evaluated the expression of CD40 in tissue samples (normal cervical tissue, cervicitis, low- and high-grade lesions, and cancer). High CD40 expression was observed in high-grade lesions (CIN II, CIN III) and especially in cervical squamous carcinomas (26,27). Interestingly, higher CD40 levels, especially in squamous carcinomas, were associated to HPV positivity (27), which was also seen in our *in silico* analysis, where CD40 expression was significantly higher in HPV-positive patients (Supplementary Figure S2). In addition, CD40 may be involved in neovascularization of cervical carcinoma, suggesting that CD40 could act as a biomarker together with VEGF to evaluate the risk of cervical cancer development or be used as a target for therapy (27). Furthermore, it was demonstrated *in vitro* using SiHa cell line that mAb anti-CD40 associated to chemotherapy has a significant therapeutic potential (28). Recent data pointed out that CD40 presents good discriminatory properties for cervical cancer, where secreted CD40 exhibits high sensitivity and specificity, making this protein an interesting target for immunotherapy combined with radiation (29).

In the present study, a positive correlation was found between transcriptional levels of CD40 and CD40L in cervical squamous carcinomas. Furthermore, high levels of CD40 and CD40L were associated with better prognosis in this type of tumor. The TCGA database showed that low CD40 and CD40L expression were associated to worse overall survival in cervical squamous carcinomas. The OS analysis (Supplementary Figure S3A and B) in patients with initial (I and II) and advanced (III and IV) tumor stages showed a worse survival curve in the group of patients with tumors in an advanced stage and low expression of CD40 and CD40L. However, the same pattern was not maintained in early stage tumors. This event probably occurred due to the different molecular mechanisms involved in cancer progression. Although this population of tumors has a higher expression of CD40, we believe that this association was due to the heterogeneity of cells in the tumor microenvironment and variability of HPV types and their relationship with cells and the microenvironment. Reinforcing this idea, the levels of its ligand, CD40L, did not change by tumor subtype (Figure 2B) and were similarly associated with OS in this population. In addition, Table 1 shows a chi-squared analysis, which is a yes-or-no scenario, whereas prognosis has other variables such as time and survival status. In this case, we have the best panoramic view. In Table 1, patients were grouped considering the median CD40L expression, and no significant association was found with clinicopathological variables. When time and vital status were added to this stratification, we observed a significant difference, where low CD40L expression was associated with worse survival. Thus, we infer that CD40L can contribute as an independent prognostic biomarker, including the observed hazard ratio of 0.54 with CI: 0.33-0.89 (Figure 3B), indicating a protective effect in patients with high CD40L expression.

Regarding CD40 and CD40L expression and cancer prognosis, a similar analysis was made, but related to breast cancer, which showed that high expression of CD40 and CD40L is also associated with better prognosis in this type of cancer (30). It has also been reported that high CD40 expression in melanomas is correlated with antitumor immune responses, better prognosis, and positive response to immune checkpoint blocking therapy or targeted therapy (5). Altogether, these data indicated that CD40 can serve as a therapeutic target for cervical cancer.

It is important to point out that *in silico* studies have some limitations that need to be considered. First, public databases mostly provide expression data based on RNA analysis. Considering that a series of post-transcriptional and post-translational events interact with mRNA, the use of such banks gives a general picture that may not correspond with the protein levels displayed by the tumor. Second, ethical regulations of some countries prevent the publication of clinical data that allow better stratification of patients, including survival analyses. Thus, although there is a large number of studies available in the GEO database, only a very small portion provides information that allows a good statistical analysis. In patients with cervical cancer with data available in the GEO database, we faced the absence of survival data and/or studies with a low number of patients. Despite the above limitations, the *in silico* study remains an extremely important tool that provided clues and ways to deepen research approaches.

In summary, CD40 was expressed in cervical cancer cell lines derived from squamous cell carcinomas, especially SiHa and SW756. Importantly, our data corroborated the Protein Atlas database, where CD40 RNA expression is found in SiHa, but not in HeLa (31). In addition, previous works also indicate that the HeLa cell line lacks CD40 expression (8,32-34). Moreover, our analysis showed a higher CD40 expression in cervical squamous carcinomas and that higher levels of CD40 and CD40L in this tumor subtype were associated with higher overall survival, suggesting better prognosis.

## Supplementary Material

Click here to view [pdf].

## References

[1] World Health Organization (WHO) (2022). Human papillomavirus (HPV) and cervical cancer. Fact sheet.

[2] Li DK, Wang W (2020). Characteristics and clinical trial results of agonistic anti-CD40 antibodies in the treatment of malignancies. Oncol Lett.

[3] Bereznaya NM, Chekhun VF (2007). Expression of CD40 and CD40L on tumor cells: the role of their interaction and new approach to immunotherapy. Exp Oncol.

[4] Argiriadi MA, Benatuil L, Dubrovska I, Egan DA, Gao L, Greischar A (2019). CD40/anti-CD40 antibody complexes which illustrate agonist and antagonist structural switches. BMC Mol Cell Biol.

[B05] Yan C, Saleh N, Yang J, Nebhan CA, Vilgelm AE, Reddy EP (2021). Novel induction of CD40 expression by tumor cells with RAS/RAF/PI3K pathway inhibition augments response to checkpoint blockade. Mol Cancer.

[6] Yan C, Richmond A (2021). Hiding in the dark: pan-cancer characterization of expression and clinical relevance of CD40 to immune checkpoint blockade therapy. Mol Cancer.

[7] Ma DY, Clark EA (2009). The role of CD40 and CD154/CD40L in dendritic cells. Semin Immunol.

[8] Tong AW, Papayoti MH, Netto G, Armstrong DT, Ordonez G, Lawson JM (2001). Growth-inhibitory effects of CD40 ligand (CD154) and its endogenous expression in human breast cancer. Clin Cancer Res.

[9] Weiss JM, Gregory Alvord W, Quiãones OA, Stauffer JK, Wiltrout RH (2014). CD40 expression in renal cell carcinoma is associated with tumor apoptosis, CD8(+) T cell frequency and patient survival. Hum Immunol.

[10] Sabel MS, Yamada M, Kawaguchi Y, Chen FA, Takita H, Bankert RB (2000). CD40 expression on human lung cancer correlates with metastatic spread. Cancer Immunol Immunother.

[11] Cerami E, Gao J, Dogrusoz U, Gross BE, Sumer SO, Aksoy BA (2012). The cBio cancer genomics portal: an open platform for exploring multidimensional cancer genomics data. Cancer Discov.

[12] Gao J, Aksoy BA, Dogrusoz U, Dresdner G, Gross B, Sumer SO (2013). Integrative analysis of complex cancer genomics and clinical profiles using the cBioPortal. Sci Signal.

[13] Lánczky A, Győrffy B (2021). Web-based survival analysis tool tailored for medical research (KMplot): development and implementation. J Med Internet Res.

[14] Chatzigeorgiou A, Lyberi M, Chatzilymperis G, Nezos A, Kamper E (2009). CD40/CD40L signaling and its implication in health and disease. Biofactors.

[15] Richards DM, Sefrin JP, Gieffers C, Hill O, Merz C (2020). Concepts for agonistic targeting of CD40 in immuno-oncology. Hum Vaccin Immunother.

[16] Vonderheide RH (2007). Prospect of targeting the CD40 pathway for cancer therapy. Clin Cancer Res.

[17] Zhang B, Wu T, Chen M, Zhou Y, Yi D, Guo R (2013). The CD40/CD40L system: a new therapeutic target for disease. Immunol Lett.

[18] Denfeld RW, Hollenbaugh D, Fehrenbach A, Weiss JM, von Leoprechting A, Mai B (1996). CD40 is functionally expressed on human keratinocytes. Eur J Immunol.

[19] Liu H, Xu J, Yang Y, Wang X, Wu E, Majerciak V (2021). Oncogenic HPV promotes the expression of the long noncoding RNA lnc-FANCI-2 through E7 and YY1. Proc Natl Acad Sci USA.

[20] Cancer Genome Atlas Research Network, Albert Einstein College of Medicine, Analytical Biological Services, Barretos Cancer Hospital, Baylor College of Medicine, Beckman Research Institute of City of Hope, et al (2017). Integrated genomic and molecular characterization of cervical cancer. Nature.

[21] Vonderheide RH, Dutcher JP, Anderson JE, Eckhardt SG, Stephans KF, Razvillas B (2001). Phase I study of recombinant human CD40 ligand in cancer patients. J Clin Oncol.

[22] Dugger K, Lowder TW, Tucker TA, Schwiebert LM (2009). Epithelial cells as immune effector cells: the role of CD40. Semin Immunol.

[23] Tong AW, Stone MJ (2003). Prospects for CD40-directed experimental therapy of human cancer. Cancer Gene Ther.

[24] Moerman-Herzog A, Nakagawa M (2015). Early defensive mechanisms against human papillomavirus infection. Clin Vaccine Immunol.

[25] Altenburg A, Baldus SE, Smola H, Pfister H, Hess S (1999). CD40 ligand-CD40 interaction induces chemokines in cervical carcinoma cells in synergism with IFN-gamma. J Immunol.

[26] Hill SC, Youde SJ, Man S, Teale GR, Baxendale AJ, Hislop A (2005). Activation of CD40 in cervical carcinoma cells facilitates CTL responses and augments chemotherapy-induced apoptosis. J Immunol.

[27] Huang Q, Qu QX, Xie F, Zhang T, Hu JM, Chen YG (2011). CD40 is overexpressed by HPV16/18-E6 positive cervical carcinoma and correlated with clinical parameters and vascular density. Cancer Epidemiol.

[28] Huang Q, Qu QX, Xie F, Hu JM, Chen YG, Zhang XG (2011). Sensitization of SiHa cell to gemcitabine by CD40 activation and its overexpression in cervical carcinoma. Med Oncol.

[29] Łaniewski P, Cui H, Roe DJ, Chase DM, Herbst-Kralovetz MM (2020). Vaginal microbiota, genital inflammation, and neoplasia impact immune checkpoint protein profiles in the cervicovaginal microenvironment. NPJ Precis Oncol.

[30] Ünver N, Yöyen Ermiş D, Weber BZ, Esendağli G (2020). Transcriptional splice variants of CD40 and its prognostic value in breast cancer. Turk J Biol.

[31] Karlsson M, Zhang C, Méar L, Zhong W, Digre A, Katona B (2021). A single-cell type transcriptomics map of human tissues. Sci Adv.

[32] Eliopoulos A, Davies C, Knox P, Gallagher N, Afford S, Adams D (2000). CD40 induces apoptosis in carcinoma cells through activation of cytotoxic ligands of the tumor necrosis factor superfamily. Mol Cell Biol.

[B33] Hakkarainen T, Hemminki A, Pereboev AV, Barker SD, Asiedu CK, Strong TV (2003). CD40 is expressed on ovarian cancer cells and can be utilized for targeting adenoviruses. Clin Cancer Res.

[34] Lin-Lee YC, Pham LV, Tamayo AT, Fu L, Zhou HJ, Yoshimura LC (2006). Nuclear localization in the biology of the CD40 receptor in normal and neoplastic human B lymphocytes. J Biol Chem.

